# Multi-mode information fusion navigation system for robot-assisted vascular interventional surgery

**DOI:** 10.1186/s12893-023-01944-5

**Published:** 2023-03-09

**Authors:** Shaoya Guan, Tianqi Li, Cai Meng, Limei Ma

**Affiliations:** 1 School of Engineers, Beijing Institute of Petrochemical Technology, Beijing, China; 2 School of Information Engineering, Beijing Institute of Petrochemical Technology, Beijing, China; 3 School of Astronautics, Beihang University, Beijing, China

**Keywords:** Navigation system, Vascular interventional surgery, Medical robotics, Interventional instrument positioning, Medical image registration

## Abstract

**Background:**

Minimally invasive vascular intervention (MIVI) is a powerful technique for the treatment of cardiovascular diseases, such as abdominal aortic aneurysm (AAA), thoracic aortic aneurysm (TAA) and aortic dissection (AD). Navigation of traditional MIVI surgery mainly relies only on 2D digital subtraction angiography (DSA) images, which is hard to observe the 3D morphology of blood vessels and position the interventional instruments. The multi-mode information fusion navigation system (MIFNS) proposed in this paper combines preoperative CT images and intraoperative DSA images together to increase the visualization information during operations.

**Results:**

The main functions of MIFNS were evaluated by real clinical data and a vascular model. The registration accuracy of preoperative CTA images and intraoperative DSA images were less than 1 mm. The positioning accuracy of surgical instruments was quantitatively assessed using a vascular model and was also less than 1 mm. Real clinical data used to assess the navigation results of MIFNS on AAA, TAA and AD.

**Conclusions:**

A comprehensive and effective navigation system was developed to facilitate the operation of surgeon during MIVI. The registration accuracy and positioning accuracy of the proposed navigation system were both less than 1 mm, which met the accuracy requirements of robot assisted MIVI.

## Background

Cardiovascular diseases are the severe causes of death globally and the number of cases suffering from these diseases is on the rise. Minimally invasive vascular intervention (MIVI), which is safe and easy for recovery, has been the state-of-the-art therapy for cardiovascular and cerebrovascular diseases. In recent years, the number of robots used in clinical surgery has increased dramatically [1, 2]. Vascular interventional surgery robots are also being developed by many institutions [3, 4].

At present, most of the navigation systems used in normal or robotic assisted MIVI are 2D navigation systems, which lack positioning informatics of the intervention instruments and 3D structure of vessels. Vascular interventional surgical robots, such as CorPath, Amigo and Niobe, all employ preoperative CTA for diagnosis and surgical planning, and use DSA images for intraoperative navigation [5–7]. Enhancing interventional visualization by integrating multi-mode intraoperative and preoperative information into a comprehensive system is of great importance of MIVI navigation [8]. Traditional vascular interventional surgery is usually guided only by DSA images [9–11], which can display the two-dimensional structure and morphology of blood vessels in real time [12]. However, patients and doctors need to be constantly exposed to the X-rays during the DSA process. Besides, the vascular structure is partially displayed in each frame with blurred boundaries and inhomogeneous distribution of contrast agent [13].

3D reconstruction of preoperative computed tomography angiograph (CTA) slices offers surgeon 3D vascular structures, while segmentation of intraoperative DSA sequences integrates vascular information in multi-frame images to provide doctors with more 2D vascular information [14–16]. 2D-3D vascular image registration combines the advantages of both preoperative CTA slices and intraoperative DSA sequences to increase visual information and reduce the use of X-rays during surgery [17–23]. Electromagnetic system (EM) as a new accurate and radiation-free positioning method has also been gradually used in the positioning of the end of interventional devices [24–27].

This study presented a navigation system combining both preoperative and intraoperative multi-mode information of MIVI. The accuracy of the navigation system has also been evaluated using clinical CTA and DSA images or a vascular model.

## Method

**Fig. 1:**
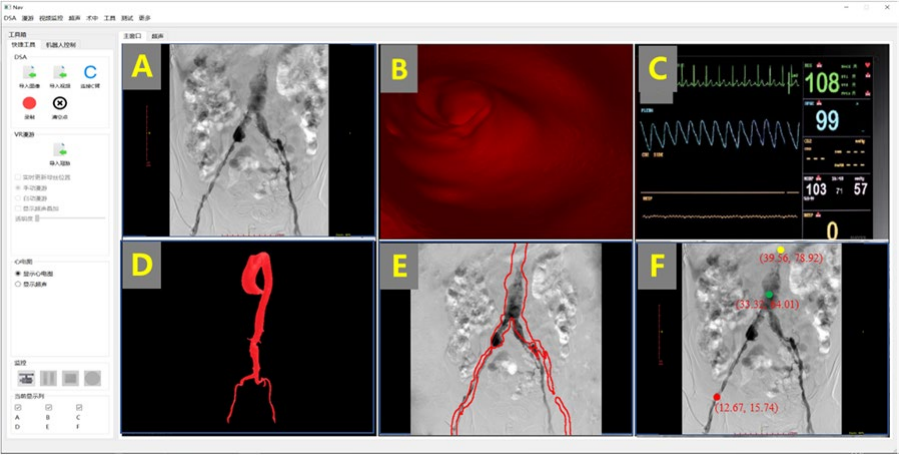
Navigation system for robot assisted MIVI. **A** was the intraoperative DSA, **B** demonstrated the internal vascular view, **C** was intraoperative physiological signal monitoring, **D** was reconstructed 3D vascular model from CTA, **E** represented the 2D-3D registration result, **F** demonstrated positioning of surgical instruments

**Fig. 2:**
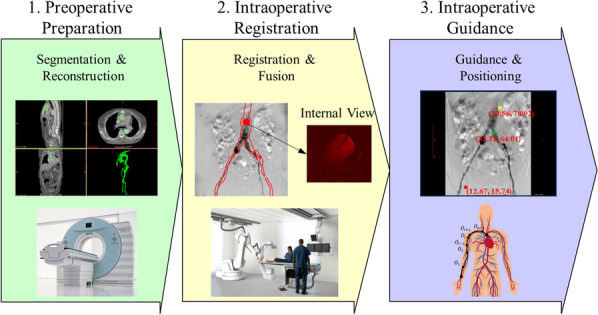
Workflow and main functions of MIFNS. Stage 1 included CTA slices segmentation and 3D vascular model reconstruction, Stage 2 demonstrated intraoperative 2D-3D image registration and fusion, Stage 3 covered surgical planning and intraoperative instrument localization

Functions of the robot assisted MIVI navigation system presented in this study, as shown in Figs. 1 and 2, include three main parts: preoperative 3D vascular reconstruction of CT slices, registration of preoperative 3D vascular model and intraoperative 2D DSA images, and intraoperative interventional instrument positioning.

3D vascular model reconstruction can be divided into three main steps: vascular segmentation from CT slices, erasing mis-segmented tissue and 3D vascular reconstruction [28]. Since the diameter of human blood vessels is difficult to quantify, the accuracy of 3D reconstruction is verified by a blood vascular model. 2D-3D registration of preoperative 3D reconstructed model and 2D intraoperative DSA images aims to find the optimal spatial transformation from a 3D model to 2D DSA images [22, 23]. The quantitative analysis of 2D-3D registration was quantified by the vascular model, while the qualitative analysis was evaluated by clinical CT and DSA images. Three evaluation criteria, mean target registration error (mTRE), mean absolute error (MAE) and dice coefficient are used in this paper to evaluate the registration result. mTRE was the mean error between registration points and target points. MAE represented the mean error between the registered and the groudtruth of the transformation parameters, mDice measured the fit between two registered images.

EM was used to measure the position of surgical instruments, and its positioning accuracy was evaluated by measuring the root mean square error (RMS) between the spatial coordinates and the EM coordinates of twenty-six catheter end positions selected on each path.

## Result

### 3D vascular model reconstruction

**Fig. 3:**
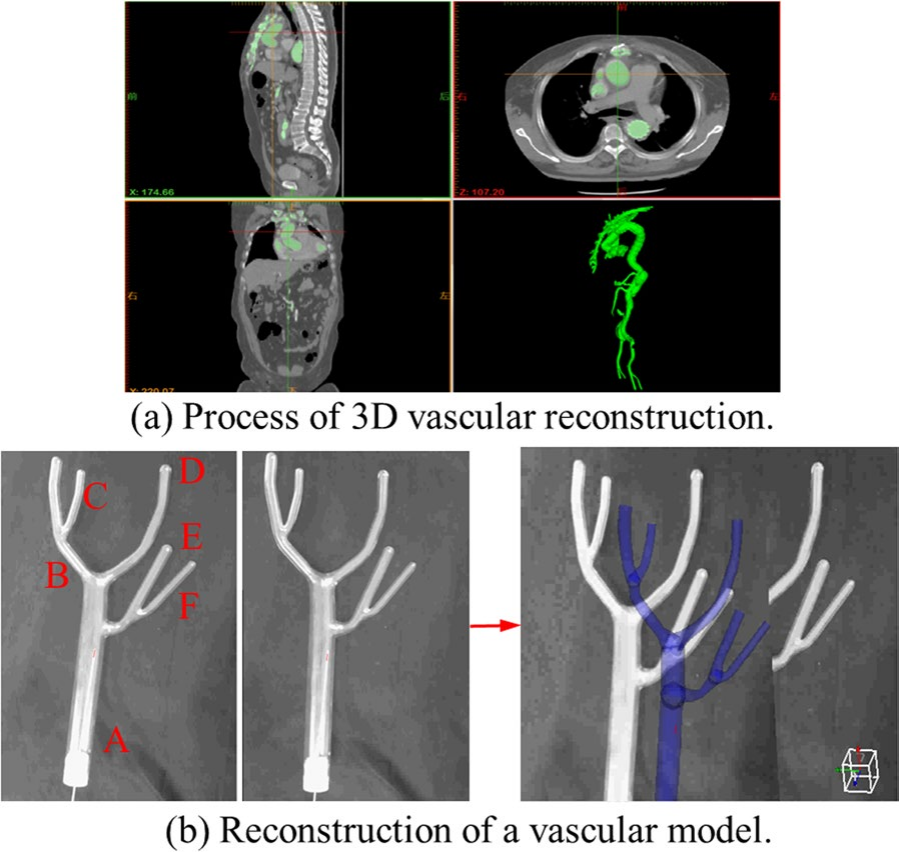
3D reconstruction of CTA slices and the vascular model

Figure 3a shows the process and results of 3D vascular reconstruction. Since the vessel diameter contained in CTA is difficult to measure due to the influence of contrast agent and blood flow, the vessel model as shown in Fig. 3b with known shape parameters is used to facilitate quantification of 3D vascular reconstruction.

The mean absolute errors (MAE) between diameters of the reconstructed model and the real model were reliable indicators to access the reconstruction accuracy. MAEs of branches A ~ F with varied diameters as shown in Fig. 3b were calculated separately and the results were shown in the following Table 1. D in Table 1 means the diameters of these branches. The MAEs demonstrated that the average reconstruction errors were all less than 1 mm, which meets the requirements for robot assisted MIVI.

**Table 1:** MAEs between diameters of the reconstruct model and the real model

	D/mm	Max/mm	Min/mm	Mean/mm
A	26	1.02	0.56	0.64
B	11	0.48	0.22	0.28
C	8	0.82	0.58	0.68
D	11	0.74	0.09	0.56
E	11	0.82	0.04	0.22
F	8	0.44	0.06	0.24

## 2D-3D image registration

**Fig. 4:**
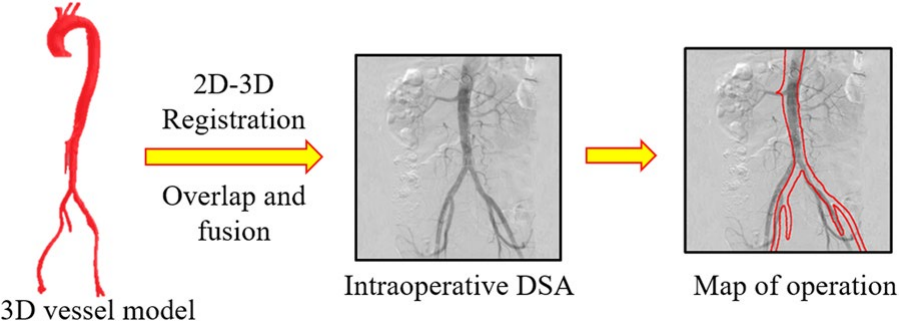
2D-3D registration of a preoperative vascular model and an intraoperative DSA

Figure 4 demonstrates the process of 2D-3D registration. The registration results directly affect the accuracy of surgical map construction. The accuracy of 2D-3D vascular image registration was evaluated by both the vascular model and clinical images. Vascular model was used to quantify the registration effect, and clinical data were used to verify the clinical application prospect of the proposed navigation system.

As shown in Table 2, the MAEs of three transformation parameters (T) were all less than 1 mm, and the MAEs of three rotation parameters (R) were smaller than or approach 0.5°. The mTREs were within the range of (0.1,0.2) that satisfied the requirement of clinical experiments. There were nine patients covered in the clinical experiments including three each with abdominal aortic aneurysm (AAA), thoracic aortic aneurysm (TAA) and aortic dissection (AD). Clinical data lacked ground truth registration result values, so mDice values as shown in Table 3 were used to measure the registration results. As shown in Table 3, mDice values of the 9 patients were all around 0.5, and there was no significant difference between the registration results of the three kinds of diseases. The main reason for the low mDice value in the registration results of clinical CTA image and DSA image is that the vascular region segmented in DSA image is affected by uneven flow of contrast agent, resulting in partial loss of blood vessels. The difference in the range of vessels covered by the two mode images leads to the low accuracy of the final registration results.

**Table 2:** Registration result of vascular model

Pos	MAE of T / mm	MAE of R /°	mTRE
1	0.62	0.35	0.11
2	0.74	0.29	0.12
3	0.54	0.36	0.11
4	0.67	0.40	0.13
5	0.93	0.31	0.17
6	0.54	0.36	0.11
Mean	0.67	0.34	0.12

**Table 3:** Registration result of clinical data

Disease	Patient	mDice	Result
AAA	1	0.45	
	2	0.52	
	3	0.47	
TAA	4	0.43	
	5	0.48	
	6	0.47	
AD	7	0.49	
	8	0.44	
	9	0.48	

## Surgical path planning and navigation accuracy

**Fig. 5:**
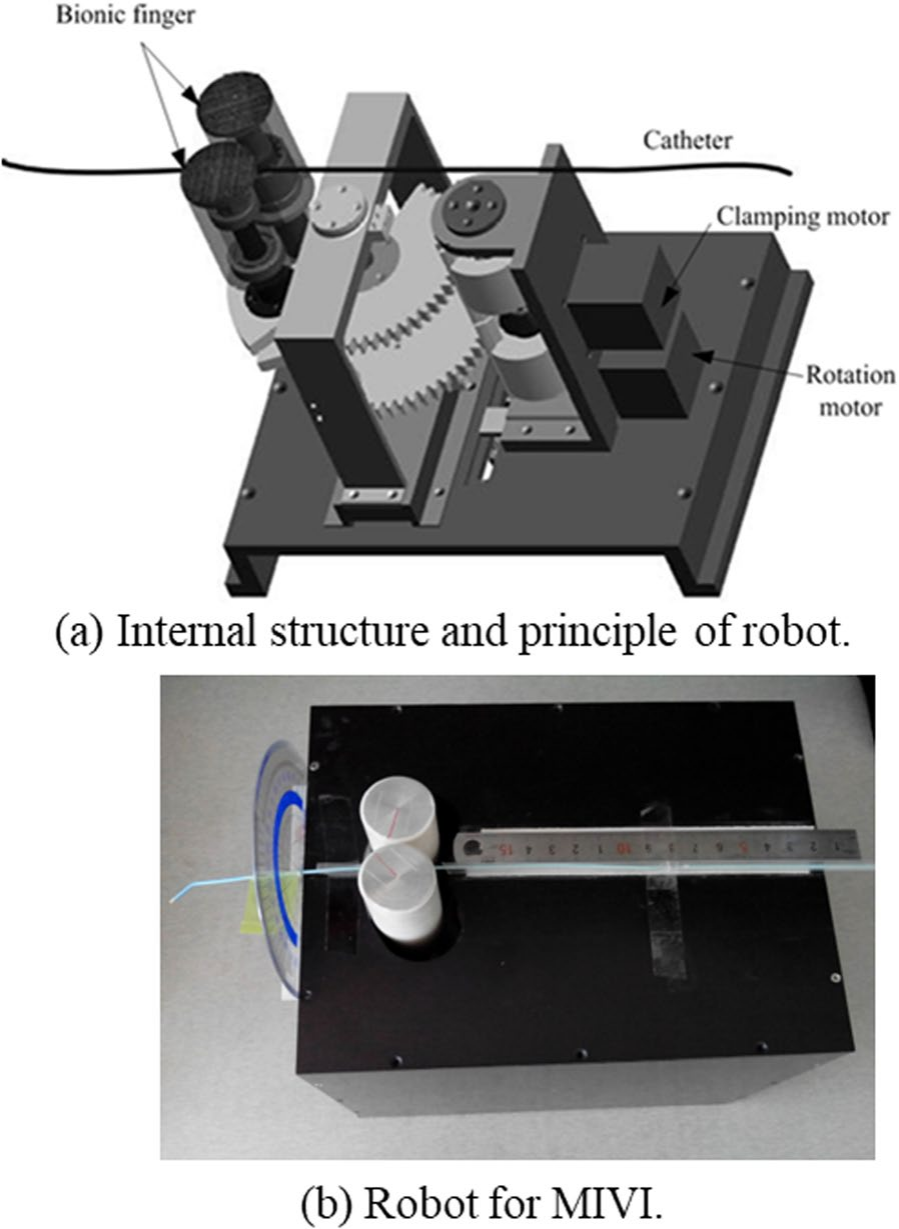
Structure and operation of the MIVI robot

**Fig. 6:**
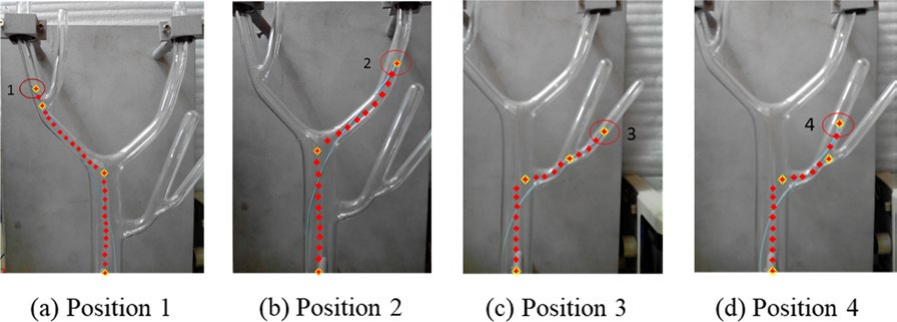
Navigation accuracy of 4 paths on the vascular model

The movement paths of the catheter from the starting point to positions 1, 2, 3 and 4 were conducted by the MIVI robot as shown in Figs. 5 and 6. The positioning accuracy of EM for the interventional device was evaluated by measuring the root mean square error (RMS) between the spatial coordinates and the EM coordinates of twenty-six catheter end positions selected on each path.

**Fig. 7:**
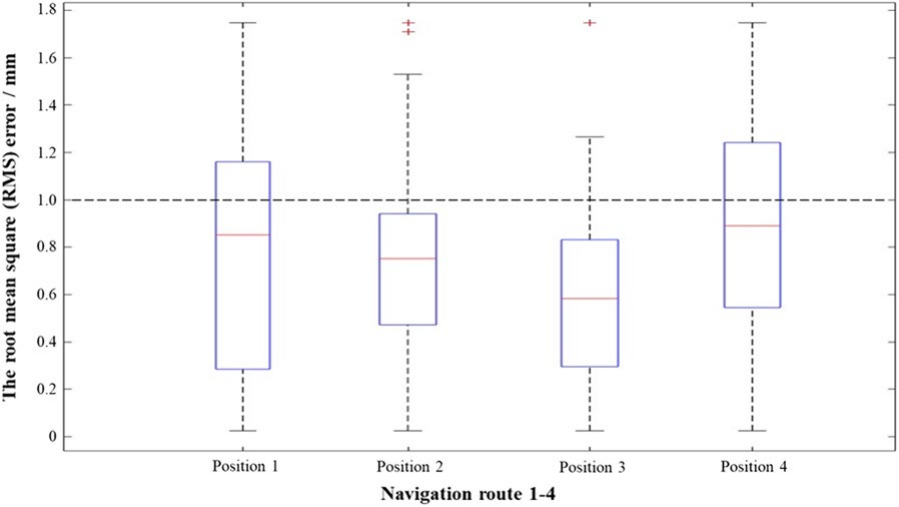
The statistical root mean square (RMS) error of 4 navigation routes

As shown in Fig. 7, the mean values of RMS were all less than 1 mm, and the maximum values were all smaller than 2 mm. The experiment results verified that the positioning accuracy of EM satisfied the requirements of clinical operations.

## Discussion

The navigation of robot assisted MIVI were successfully completed on the vascular model with high accuracy and less contrast injection. The 2D-3D image registration combining both the advantages of CTA and DSA, makes it possible to simultaneously visualize the preoperative and intraoperative images. Accurate 3D reconstruction of blood vessels and positioning of surgical instruments ensure the operation accuracy of robot-assisted surgery and improve the safety of surgery.

First of all, 3D vascular models containing sufficient vascular shape and structure information were reconstructed from preoperative CTA slices, which were the foundation of three-dimensional navigation. The accuracy of vascular 3D reconstruction was quantified by vascular model to verify that it satisfied the clinical requirements. Meanwhile, 2D-3D vascular image registration combined the advantages of preoperative 3D vascular model and intraoperative DSA images to increase the real-time performance of the navigation system. The registration result offers surgeon a global vascular map which only needs a clear DSA sequence during operation. The reduction of the demand for DSA sequence decreases the use of contrast agent during the operation. Although the performance of 2D-3D registration on clinical data was not as satisfied as that on vascular models, it provided doctors with a static but intuitive map of vessels. Moreover, surgical path planning helped doctors or robotics select the optimal surgical route to shorten the surgical time and improve the surgical safety. Finally, the EM system was used to track and locate the surgical instruments by integrating the multi-mode information of the navigation system. The average positioning accuracy was less than 1 mm, which evaluated the accuracy and clinical application prospect of the navigation system.

## Conclusion

The multi-mode information fusion navigation system proposed in this paper for TIVI combined multi-modal medical information during both preoperative and intraoperative operation to provide comprehensive and intuitive visual navigation for doctors or robots. The main functions of the navigation system were evaluated by sufficient experiments on clinical data and vascular models to verify that they satisfied the clinical applications. The navigation system also needs further improvements to provide more accurate navigation information for doctors and robots. For example, the clinical application of the navigation system is only partially verified in this paper. In future studies, more clinical animal and human experiments will be covered in our research to verify the function and accuracy of the navigation system.

## Data Availability

The datasets analyzed during the current study are not publicly available due to respect the participants’ rights to privacy and to protect their identity, but are available from Shaoya Guan on reasonable request.
